# Following the Reaction of Heteroanions inside a {W_18_O_56_} Polyoxometalate Nanocage by NMR Spectroscopy and Mass Spectrometry**

**DOI:** 10.1002/anie.201502295

**Published:** 2015-05-26

**Authors:** Qi Zheng, Laia Vilà-Nadal, Christoph Busche, Jennifer S Mathieson, De-Liang Long, Leroy Cronin

**Affiliations:** WestCHEM, School of Chemistry, The University of Glasgow Glasgow G12 8QQ (UK) E-mail: Lee.Cronin@glasgow.ac.uk Homepage: http://www.croninlab.com

**Keywords:** cluster compounds, electronic structure, NMR spectroscopy, phosphorus, polyoxometalates

## Abstract

By incorporating phosphorus(III)-based anions into a polyoxometalate cage, a new type of tungsten-based unconventional Dawson-like cluster, [W_18_O_56_(HP^III^O_3_)_2_(H_2_O)_2_]^8−^, was isolated, in which the reaction of the two phosphite anions [HPO_3_]^2−^ within the {W_18_O_56_} cage could be followed spectroscopically. As well as full X-ray crystallographic analysis, we studied the reactivity of the cluster using both solution-state NMR spectroscopy and mass spectrometry. These techniques show that the cluster undergoes a structural rearrangement in solution whereby the {HPO_3_} moieties dimerize to form a weakly interacting (O_3_PH⋅⋅⋅HPO_3_) moiety. In the crystalline state the cluster exhibits a thermally triggered oxidation of the two P^III^ template moieties to form P^V^ centers (phosphite to phosphate), commensurate with the transformation of the cage into a Wells–Dawson {W_18_O_54_} cluster.

Polyoxometalates (POMs) are a unique class of metal oxide anions exhibiting a large variety of structural versatility and interesting physical properties,[1–3] as well as the ability to show variable redox potentials.[4] Wells–Dawson-type (WD) clusters with a general formula of [W_18_O_54_(XO_4_)_2_]^*n*−^ (X=S^VI^, P^V^, Si^IV^, etc.) are one of the most widely investigated families of molecules in the POM family.[5] WD clusters are described as rigid nanocages {W_18_O_54_} in which two tetrahedral heteroanions {XO_4_} are embedded and their electrochemistry has been extensively examined.[6] However, the properties of WD clusters are mainly attributed to the multiple redox states of the rigid metal oxide framework. We have successfully manipulated the redox behavior of WD clusters by introducing electronically active heteroatoms into the metal oxide shell. In this respect, a series of novel Dawson-like clusters with redox-active templating anions have been successfully isolated, for example, {W_18_O_57_(Te^IV^O_3_)}, {W_18_O_59_(I^V^O_3_)}, {M_18_O_54_(XO_3_)_2_} (X=S^IV^ or Se^IV^, M=Mo or W) and {W_18_O_56_(XO_6_)} (X=Te^VI^, I^VII^ and W^VI^).[7] All of these clusters exhibit unprecedented intramolecular electron-transfer features that are modulated directly by the enclosed anions. The so-called “Trojan horse”-type cluster [W_18_O_56_(S^IV^O_3_)_2_(H_2_O)_2_]^8−^ is a remarkable compound.[7a] It differs from the classic WD cluster by its unique structural features whereby 4 of the 18 tungsten atoms have two terminal ligands (2 oxo and 2 aqua) on the {W_18_} shell. In fact, the ability of these activated unconventional POM compounds to store electrons was recently used in the field of electronic storage devices: in 2014 we reported the successful integration of a Dawson-like cluster [W_18_O_54_(SeO_3_)_2_]^4−^ into a flash memory architecture.[8] This result drove us to the development of a new heteroatom-templated functional system. However, despite this work[7, 8] we are yet to obtain direct spectroscopic evidence demonstrating the reactivity of heteroanions “trapped” within a molecular metal oxide polyoxometalate nanocage.

Herein, we report a system that allows us to directly probe the transformation of reactive templates within a polyoxometalate nanocage using NMR spectroscopy and mass spectrometry. To do this we designed and synthesized a P^III^-embedded polyoxotungstate [W_18_O_56_(HP^III^O_3_)_2_(H_2_O)_2_]^8−^ (**1 a**; Figure 1) in which two phosphite anions [HPO_3_]^2−^ were trapped inside a {W_18_} nanocage. This material was isolated as (C_2_H_8_N)_8_[W_18_O_56_(HP^III^O_3_)_2_(H_2_O)_2_]⋅20 H_2_O (**1**). The isolation of this nanocage demonstrates that it is possible to introduce a redox-active phosphorus heteroatom into a Dawson-like nanocage. In contrast, the only reported phosphorus-templated Dawson compound contains non-redox-active P^V^ centers.[9] Cluster **1** was specifically targeted and was synthesized by a one-pot reaction of sodium tungstate (Na_2_WO_4_) and phosphorous acid (H_3_PO_3_) at pH 2.8 in the presence of dimethylamine hydrochloride (DMA) as a structure-directing agent. Subsequent crystallization, over a two-week period, yielded crystals of **1**, which were obtained in the monoclinic system with space group *P*2_1_/*m*. The X-ray crystal structure of **1** revealed that it was an isostructure of the “Trojan horse”-type cluster [W_18_O_56_(S^IV^O_3_)_2_(H_2_O)_2_]^8−^ previously reported by us.[7a] The compound had a *C*_2*v*_-symmetric {W_18_O_56_} cluster shell in which two [HPO_3_]^2−^ units were embedded with an average P–O distance of 1.55(5) Å and an average (P)O–W distance of 2.44(9) Å. The key structural feature of this type of cluster is the orientation of the embedded template moieties, where the two {HPO_3_} moieties are tilted and are connected to only seven of the nine W centers in the {W_9_(HPO_3_)} fragment. The two unconnected W centers have two terminal oxo ligands (or one oxo plus one aqua ligand), resulting in a slightly distorted octahedral coordination geometry. Furthermore, compared with the two previously reported {W_18_O_56_} compounds having [SO_3_]^2−^ and [SeO_3_]^2−^ embedded in the cluster shell, the major difference in the structure of **1 a** is the protonation of the heteroatoms. The result of this protonation is that there are two hydrogen atoms located in the center of the cluster which also gives us an additional reactive and spectroscopic handle to explore transformations within the cage.

**Figure 1:**
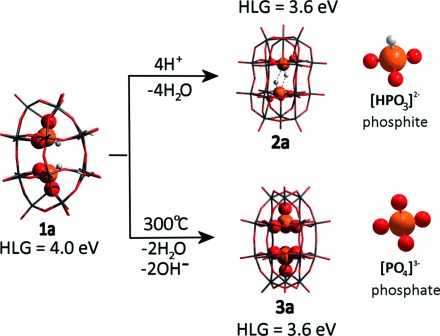
Structure of “Trojan horse”-type cluster [W_18_O_56_(HP^III^O_3_)_2_(H_2_O)_2_]^8−^ (1 a) in solution, peanut-like Dawson cluster [W_18_O_54_(HP^III^O_3_)_2_]^4−^ (2 a; proposed structure), and the classic Wells–Dawson cluster [W_18_O_54_(P^V^O_4_)_2_]^6−^ (3 a). Energy values for the HOMO–LUMO gap (HLG) for compounds 1 a, 2 a, and 3 a are given in eV, and their frontier orbitals are shown in Figure S13. Atom colors: O=red; P=orange; W=dark gray; H=light gray.

Based on our previous study of “Trojan horse”-type molecules, {W_18_O_56_} is a very dynamic molecular system that is easily transformed to a peanut-shaped {W_18_O_54_} cluster, driven by cation exchange and the loss of water ligands.[7b] One significant feature of the {W_18_O_54_} clusters is the central alignment of two heteroatoms in a face-to-face orientation, leading to a shorter distance between the two heteroatoms than in {W_18_O_56_}. The transformation is therefore favorable for the formation of heteroatom interactions within metal oxide cages. Inspired by this prospect, density functional theory (DFT) calculations were conducted to investigate the feasibility of transformation of the phosphite-embedded cluster **1**. Molecular orbital diagrams (see the Supporting Information, Figure S13) show the classic HOMO (on the *p*-oxo fragments) and LUMO (on the d metal fragments) delocalization common to most POMs.[10] The value of the HOMO–LUMO gap (HLG) is a good indication of the cluster stability. In this case, for clusters **1 a** (4 eV) and peanut-shaped [W_18_O_54_(HP^III^O_3_)_2_]^4−^ (**2 a**; 3.6 eV), they are in the same range as the classic P^V^-containing Wells–Dawson cluster **3 a** (3.6 eV; see Figure 1). This agreement further demonstrates the stability of the phosphite cluster and suggests that the rearrangement of Trojan-horse cluster **1 a** to form peanut-shaped cluster **2 a** is feasible. Guided by the theoretical insights, we have conducted high-resolution ESI-MS experiments to examine the properties of the clusters in solution (Figure S2). All major peaks in the mass spectra showed consistent results implying a transformation of {W_18_O_56_} cluster **1 a** to form HPO_3_-embedded {W_18_O_54_} clusters **2 a**. Major signals in the mass spectra (with calculated values and anion compositions in parentheses) were detected at *m*/*z* 2189.76 (2189.92; [(DMA)HW_18_(HPO_3_)_2_O_54_]^2−^), 2178.25 (2178.39; [NaHW_18_(HPO_3_)_2_O_54_]^2−^), 2167.25 (2167.39; [H_2_W_18_(HPO_3_)_2_O_54_]^2−^), 1444.49 (1444.59; [H_1_W_18_(HPO_3_)_2_O_54_]^3−^), and 1083.12 (1083.19; [W_18_(HPO_3_)_2_O_54_]^4−^).

The structural transformation of the cluster in aqueous solution was unambiguously confirmed by NMR spectroscopy (Figure 2). The ^31^P NMR spectrum exhibits a doublet, arising as a result of the proton coupling with phosphorus (^1^*J*_PH_=781 Hz), centered at *δ*=9.0 ppm (Figure 2 a). Coupling constant ^1^*J*_PH_ values are frequently in the range of 400–1000 Hz,[11] and the ^1^*J*_PH_ value is mainly sensitive to the hybridization of the P–H bond.[12] The ^1^H NMR spectrum is similar, showing a doublet with the same coupling constant (Figure S1). This coupling is further corroborated by decoupling NMR experiments, where the two signals of the doublet merged to form a singlet (Figure 2 b). This change suggests that the splitting of the signal is indeed as a result of the protons attached to the phosphorus nuclei; the two phosphorus centers in the {W_18_} cage are chemically equivalent, which is consistent with the *C*_2*v*_ symmetry of the clusters, as determined by crystallography. The detection of such a large {^31^P}–{^1^H} coupling constant within an inorganic molecule is interesting and the 4.4 ppm downfield shift (from *δ*=4.6 to 9 ppm) of the signal attributable to the P center provides good evidence for encapsulation. Each signal is broadened and further split giving a doublet with a *J*_PH_ value of 8.84 Hz with the same peak pattern also being detected in the ^1^H NMR spectrum. The measurement of this peak pattern is consistent with the formation of intramolecular P–H⋅⋅⋅P hydrogen-bonded interactions between the phosphorus nuclei in each half {W_9_} cage, as depicted in Figure 2.

**Figure 2:**
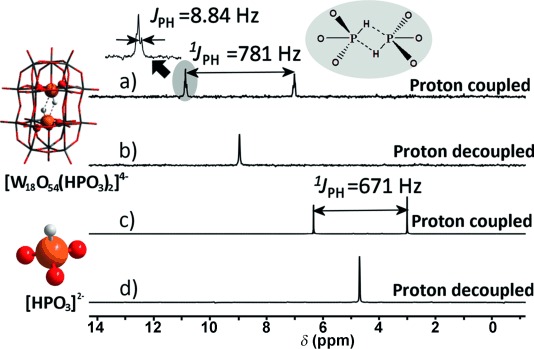
a, c) Proton-coupled and b, d) proton-decoupled ^31^P NMR spectra of cluster 1 a [W_18_O_56_(HP^III^O_3_)_2_(H_2_O)_2_]^8−^ (a, b) and phosphorous acid [HPO_3_]^2−^ (c, d) in solution showing their coupling constants (1 a: ^1^*J*_PH_=781 Hz; [HPO_3_]^2−^: 671 Hz) and chemical shifts (1 a: *δ*=9.0 ppm; [HPO_3_]^2−^: 4.6 ppm).

Given the nature of the thermodynamic instability of sulfite-containing “Trojan horse”-type clusters, we hypothesized that the same temperature sensitivity would apply to the phosphite system. We therefore undertook precise temperature-controlled studies which showed remarkable differences compared to the sulfite system. The sulfite system itself transformed to the blue {W^VI^_14_W^V^_4_O_54_(SO_4_)_2_} species when heated to over 400 °C, essentially trapping the electrons on the cluster shell when the sulfite was oxidized to sulfate.[13] However, the HPO_3_-templated colorless compound **1** (at RT) turned dark blue (at temperatures greater than 200 °C), and subsequently turned yellow in air. From this observation, we hypothesize that the {W_18_} underwent a redox cycle, that is, W^VI^(colorless)→W^V^(dark blue)→W^VI^(colorless); whereas the colorless {HP^III^O_3_} moiety experienced an oxidation reaction to form a {P^V^O_4_} fragment (yellow).

^31^P NMR spectroscopic analysis was carried out to identify the oxidation pathway of the phosphorus species (Figure 3). Solid samples from each temperature-controlled experiment were collected under nitrogen and were dissolved in D_2_O at room temperature. The resulting NMR spectrum indicated the presence of the P–H moiety and the magnitude of the P–H coupling constant was found to decrease slightly upon increasing the temperature to 200 °C. However, upon heating to around 300 °C, the resonance signals for the yellow product showed a dramatic shift to higher field. The singlets which appear at *δ*=−13.0 ppm are identical to those for the α isomers of the Wells–Dawson cluster {P^V^_2_W_18_O_62_}.[14] Additionally, the ^31^P NMR (with decoupled proton signals) and ^1^H NMR spectra (Figure S3–5) are consistent with the oxidation of the phosphite to phosphate. Rather unexpectedly, from 220 °C to 280 °C the spectra of the dark-blue compounds showed well-resolved doublets of equal intensity at *δ*=13.1 ppm and 9.1 ppm which have not undergone further splitting. These signals indicated the dissociation of the diphosphite (O_3_PH⋅⋅⋅PO_3_H) interactions with only one {HP^III^O_3_} moiety remaining. This results in the formation of a dark-blue reduced cluster with a mixed-valence tungstate shell, which is evidenced by electron paramagnetic resonance spectroscopy (Figure S6). In addition, the new resonance signal at *δ*=−10.3 ppm suggests the presence of another phosphorus species inside the molecular cage. Combined with the observation of the blue compound, we hypothesized the structural transformation from [W_18_O_56_(HPO_3_)_2_(H_2_O)_2_]^8−^ **1 a** to [W_18_O_54_(PO_4_)_2_]^4−^ **3 a** occurred through an intermediate [W^VI^_18-*x*_W^V^_*x*_O_55_(HPO_3_)(PO_4_)]^*n*−^ species. This structural rearrangement is highly unusual given that a half peanut-shaped {W_9_(XO_3_)} and a half Wells–Dawson-type {W_9_(XO_4_)} fragment have been combined, a combination which should give rise to a structurally unfavorable cluster. A DFT calculation exploring this geometry showed a reasonable HLG value at 3.5 eV (Figure S13). Another explanation for this pathway would be that the thermal activation (>200 °C) triggers the cleavage of the P–H bond of the phosphite moieties to form two phosphite radicals (^.^PO_3_), which then spontaneously disproportionate into phosphite and orthophosphate. However, the intermediate oxidation state (P^IV^) could not be detected in the EPR experiments thus far.

**Figure 3:**
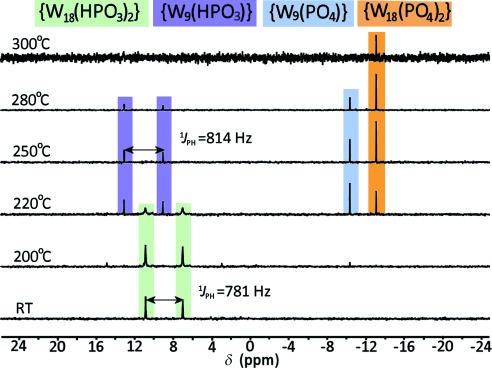
^31^P NMR spectra of samples of the “Trojan horse”-type cluster 1 a collected from the variable-temperature experiments (RT to 300 °C) and dissolved in D_2_O.

To verify the hypothesis and to follow the structural transformation directly, ESI-MS experiments were conducted using cluster **1** heated at variable temperatures and dissolved in a mixture of water and acetonitrile (5 %:95 %; Figure 4). At *m*/*z*=1087.2 a signal corresponding to [W_18_O_54_(HP^III^O_3_)(P^V^O_4_)H]^4−^ was detected directly in the solution of the blue compound. Additionally, the intensity of signals assigned to [W_18_O_54_(P^V^O_4_)_2_H]^4−^ at *m*/*z*=1091.1 increased in the higher temperatures samples (Figure 4). Similar patterns were also detected for the 5-, 3-, and 2- charge envelopes (Figure S7–9, Table S3). From the combination of our crystallographic, theoretical, and spectroscopic measurements we have been able to form a complete picture of the first P^III^-containing POM cluster reactivity and propose a potential mechanism for the temperature-dependent, solid-state structural rearrangement of the “Trojan horse”-type {W_18_O_56_} to the Wells–Dawson {W_18_O_54_} cluster (Figure 5).

**Figure 4:**
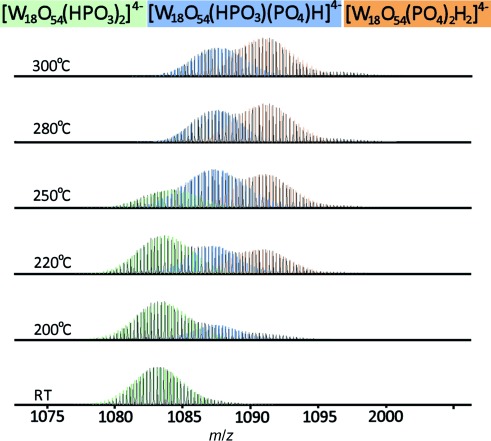
ESI mass spectra showing the transformation from the Dawson-like [W_18_O_54_(HPO_3_)_2_]^4−^ to the Wells–Dawson-type cluster [W_18_O_54_(PO_4_)_2_]^6−^ through the intermediate [W_18_O_54_(HPO_3_)(PO_4_)H]^4−^_._

**Figure 5:**
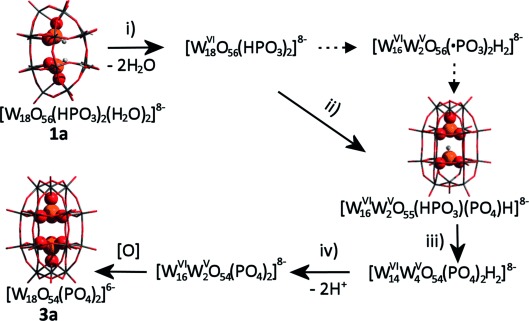
Proposed pathway for temperature-dependent solid-state structural transformation of 1 a to form 3 a. i) Upon heating over 100 °C, crystals of cluster 1 a [W^VI^_18_O_56_(HP^III^O_3_)_2_(H_2_O)_2_]^8−^ are dehydrated and transformed to an intermediate species [W^VI^_18_O_56_(HP^III^O_3_)_2_]^8−^. ii) At around 220 °C, the oxidation of the heteroatomic moiety {HPO_3_} begins. At this point two moieties {HP^III^O_3_} and {P^V^O_4_} coexist within the cluster, also reducing the cluster shell to give a blue intermediate [W^VI^_16_W^V^_2_O_55_(HP^III^O_3_)(P^V^O_4_)H]^8−^. This intermediate is further reorganized into [W_18_O_54_(HPO_3_)(PO_4_)H]^8−^ in solution, as detected in the ESI-MS experiments. iii) Over 280 °C, further oxidation of the remaining P^III^ centers is favored, giving rise to the formation of the mixed-valence blue-colored Wells–Dawson cluster [W^VI^_14_W^V^_4_O_54_(P^V^O_4_)_2_H_2_]^8−^. iv) The elimination of protons, by loss of water, is the final step to obtain the fully oxidized yellow Wells–Dawson compound 3 a [W^VI^_18_O_54_(P^V^O_4_)_2_]^6−^.

In summary, we have discovered a phosphorus(III) heteroatom-templated polyoxotungstate compound, [W_18_O_56_(HP^III^O_3_)_2_(H_2_O)_2_]^8−^, with two embedded {HPO_3_} moieties in a “Trojan horse”-type {W_18_} nanocage. In addition to X-ray crystallographic analysis, we have characterized these polyanions with NMR spectroscopy and have confirmed the protonation of the templating phosphite anions. In the solution state, we detected an unusual inter-phosphite (O_3_PH⋅⋅⋅PO_3_H) interaction due to the structural transformation to a peanut-shape {W_18_O_54_} nanocage. In the solid state, we were able to show that a temperature-dependent intramolecular redox reaction and structural rearrangement from [W_18_O_56_(HPO_3_)_2_(H_2_O)_2_]^8−^ to [W_18_O_54_(PO_4_)_2_]^4−^ occurs. This rearrangement appears to proceed via an intermediate two-template-containing cluster, that is, a pyramidal {HPO_3_} and a tetrahedral {PO_4_} moiety, which was detected directly through NMR spectroscopy and mass spectroscopy and confirmed by DFT calculations. In future work, we will focus on expanding this family of P^III^-containing polyoxometalates as well as investigating the electrochemical and photochemical properties in addition to attempting to form, trap, and detect the P^IV^ radicals.

## Experimental Section

Preparation of **1**: Na_2_WO_4_⋅2 H_2_O (5.5 g, 16.7 mmol), H_3_PO_3_ (0.10 g, 1.22 mmol), and dimethylamine hydrochloride (DMA) (2.0 g, 13.0 mmol) were dissolved in water (15 mL). Diluted hydrochloric acid was added dropwise under stirring, and the pH value was adjusted to 2.8. The solution was heated with stirring until it become cloudy. Upon cooling down to room temperature, a white powder formed and was removed by filtration. Colorless block crystals of **1** were isolated from the filtrate after two weeks. These crystals were collected by soaking the solid using the filtered mother liquid to remove further white powder. Yield: 4.4 % (based on W). Elemental analysis calcd (%) for C_16_H_110_N_8_W_18_P_2_O_84_ (**1**): W 64.5, P 1.21, H 2.16, C 3.74, N 2.18; found: W 63.2, P 1.15, H 1.42, C 3.79, N 2.10.

Computational method: Geometry optimizations were performed using the B3LYP method as implemented in the TURBOMOLE V6.3.1 3 package.[15] For these calculations, the TZVP basis set was used on all atoms. To allow for solvation effects, the conductor-like screening model (COSMO) method was used with ionic radii of the atoms, which define the dimensions of the cavity surrounding the molecule, are chosen to be (in Å) 2.23 for W, 1.72 for O, 2.11 for P, 1.3 for H.

## Supporting Information

As a service to our authors and readers, this journal provides supporting information supplied by the authors. Such materials are peer reviewed and may be re-organized for online delivery, but are not copy-edited or typeset. Technical support issues arising from supporting information (other than missing files) should be addressed to the authors.

miscellaneous_information
