# Supplementary material for: Following the Reaction of Heteroanions inside a {W18O56} Polyoxometalate Nanocage by NMR Spectroscopy and Mass Spectrometry
Source: Angew Chem Int Ed Engl. 2015 May 26;54(27):7895–9. doi: 10.1002/anie.201502295 (PMC4557056; doi:10.1002/anie.201502295)
Supplement: Supplementary file 1 — miscellaneous_information [file anie0054-7895-sd1.pdf]

Angewandte Chemie
Eine Zeitschrift der Gesellschaft Deutscher Chemiker

Supporting Information

Following the Reaction of Heteroanions inside a $\{W_{18}O_{56}\}$ Polyoxometalate Nanocage by NMR Spectroscopy and Mass Spectrometry**

*Qi Zheng, Laia Vilà-Nadal, Christoph Busche, Jennifer S. Mathieson, De-Liang Long, and Leroy Cronin**

anie_201502295_sm_miscellaneous_information.pdf

Table of Contents S1

1. Materials S2
2. Instrumentation S2
3. Crystallographic data S4
4. NMR data S5
5. Mass Spectrometry data S7
6. EPR spectra S10
7. Infrared spectra S11
8. PXRD spectra S12
9. TGA and DSC S13
10. Theoretical calculations S14
11. References S16

1. Materials

Reagent-grade chemicals were obtained from Aldrich Chemical Company Ltd. and Alfa Aesar, and used without further purification.

2. Instrumentation

Crystallography: Suitable single crystals were selected and mounted onto the end of a thin glass fibre using Fomblin oil. X-ray diffraction intensity data were measured at 150(2) K on a Bruker Apex II Quasar diffractometers using MoKα [λ = 0.71073 Å]. Structure solution and refinement were carried out with SHELXS-97[1] and SHELXL-97[2] via WinGX[3]. Corrections for incident and diffracted beam absorption effects were applied using analytical methods[4]. The structure has been deposited to CCDC with the entry number CCDC-1052190.

Mass Spectrometry:

Measurements were carried out at 180 °C in water using a Bruker MaXis Impact instrument. The calibration solution used was Agilent ESI L low concentration tuning mix solution, Part No. G1969 - 85000, enabling calibration between approximately 50 m/z and 2000 m/z. Samples were dissolved in a water/acetonitrile mixture (5%:95%) and introduced into the MS at a dry gas temperature of 180 °C. The ion polarity for all MS scans recorded was negative, with the voltage of the capillary tip set at 4500 V, end plate offset at −500 V, funnel 1 RF at 400 Vpp and funnel 2 RF at 400 Vpp, hexapole RF at 200 Vpp, ion energy 5.0 eV, collision energy at 15 eV, collision cell RF at 2100 Vpp, transfer time at 120.0 μs, and the pre-pulse storage time at 20.0 μs.

^{1}H and ^{31}P Nuclear Magnetic Resonance Spectroscopy: ^{1}H and ^{31}P NMR spectroscopy were recorded on a Bruker DPX 500 spectrometer using the solvent signal as internal standard. All samples were prepared by dissolving the clusters in D_2O.

EPR measurements: Temperature-dependent X-Band EPR spectra were recorded on a Bruker Elexsys 500E spectrometer with a cylindrical TE_{011} cavity as approximately 1 $\times 10^{-4}$ M frozen solution (liquid nitrogen temperatures) in D_2O.

Fourier-transform infrared (FT-IR) spectroscopy: The sample was prepared as a KBr pellet and the FT-IR spectrum was collected in transmission mode in the range of 400-4000 cm^{-1} using a JASCO FT-IR 4100 spectrometer. Wavenumbers are given in cm^{-1}.

Microanalysis: Carbon, nitrogen and hydrogen content were determined by the microanalysis services within the Department of Chemistry, University of Glasgow using an EA 1110 CHNS, CE-440 Elemental Analyzer.

Thermogravimetric Analysis (TGA): Thermogravimetric analysis was performed on a TA Instruments Q 500 Thermogravimetric Analyzer under nitrogen flow at a typical heating rate of 10°C min^{-1}.

Differential Scanning Calorimetric Analysis (DSC): DSC analysis was performed on a TA Instruments Q 200 calorimeter under nitrogen flow at a typical heating rate of 10°C min^{-1}.

3. Crystallographic data

Table S1: Crystallographic Details for Compound **1**

Compound	**1**
Empirical formula	C16 H110 N8 O84 P2 W18
Formula weight	5130.35
Temperature (K)	150(2)
Wavelength (Å)	0.71073
Crystal system	Monoclinic
Temperature (K)	150
Space group	P 21/m
a (Å)	13.8991(6)
b(Å)	21.4828(9)
c (Å)	15.9306(7)
α (deg)	90
β (deg)	91.749(2)
γ (deg)	90
Volume ($Å^3$)	4754.5(4)
Z	2
Density (calculated) (Mg/m^3)	3.584
Absorption coefficient (mm^{-1})	21.822
F(000)	4592
Crystal size (mm)	0.191 x 0.091 x 0.063
Theta range for data collection (deg)	1.896 to 25.999
Limiting indices	-17<=h<=17, -26<=k<=26, -19<=l<=19
Reflections collected / unique	66374 / 9587 [R(int) = 0.0646]
Completeness to theta = 25.242	99.9 %
Absorption correction	Empirical
Max. and min. transmission	1.000 and 0.505
Refinement method	Full-matrix least-squares on F2
Data / restraints / parameters	9587 / 9 / 530
Goodness-of-fit on F^2	1.145
Final R indices [I>2δ (I)]	R1 = 0.0413, wR2 = 0.0964
R indices (all data)	R1 = 0.0679, wR2 = 0.1198
Largest diff. peak and hole (e. $Å^{-3}$)	2.83 and -1.83

4. NMR data

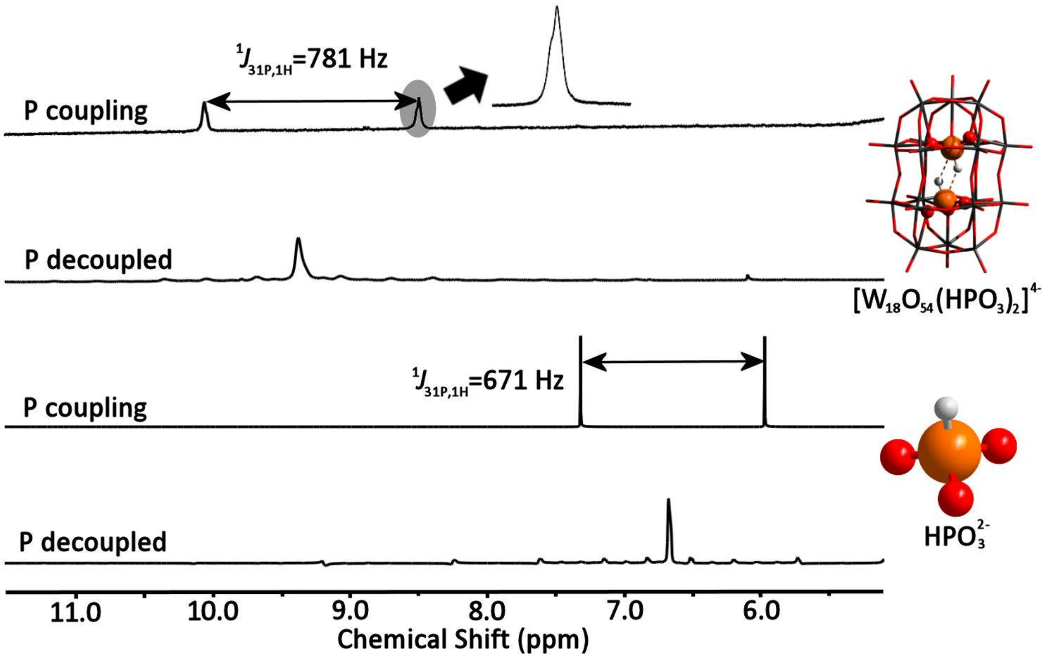

Figure S1. ^{1}H NMR spectra of cluster **1a** in D_2O with ^{31}P coupled and decoupled.

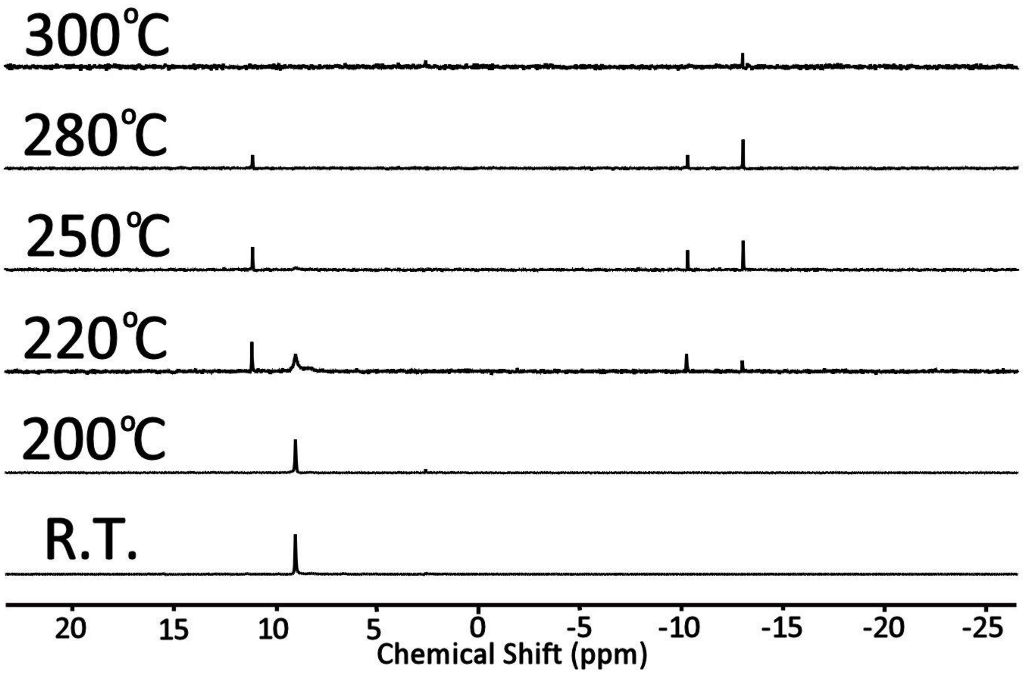

Fig S3. ^{31}P NMR with ^{1}H decoupled of the "Trojan Horse"-type cluster **1a** heated from R.T. to 300°C.

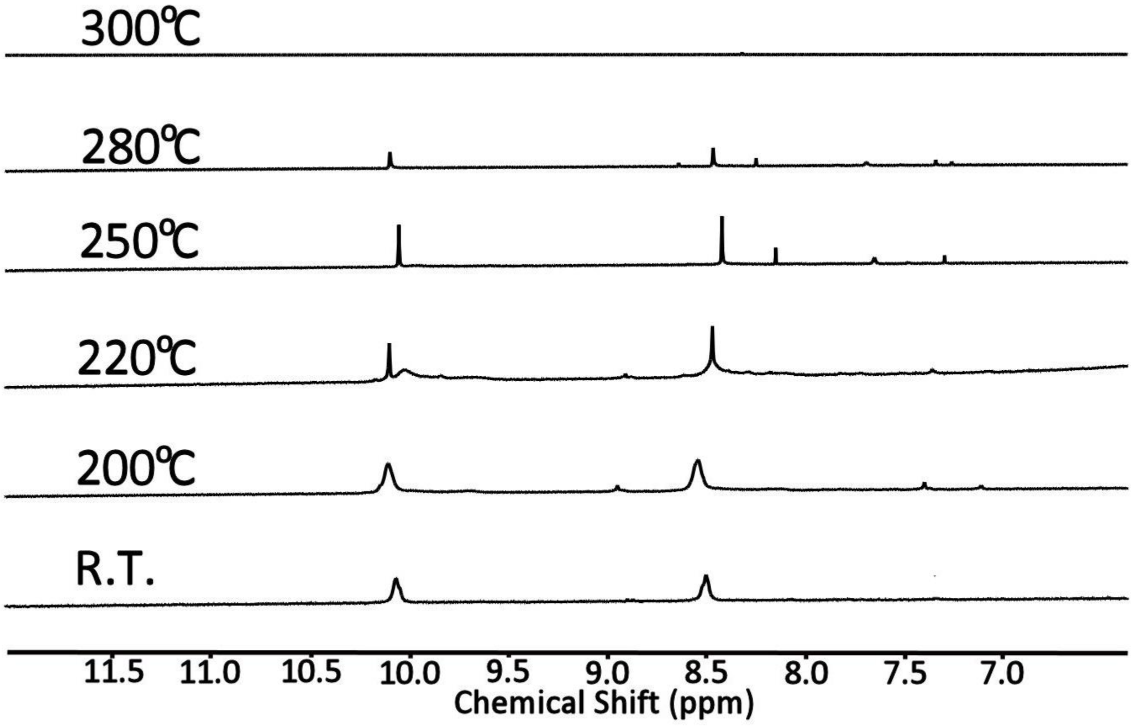

Fig S4. ^{1}H NMR of the "Trojan Horse"-type cluster **1a** heated from R.T to 300°C.

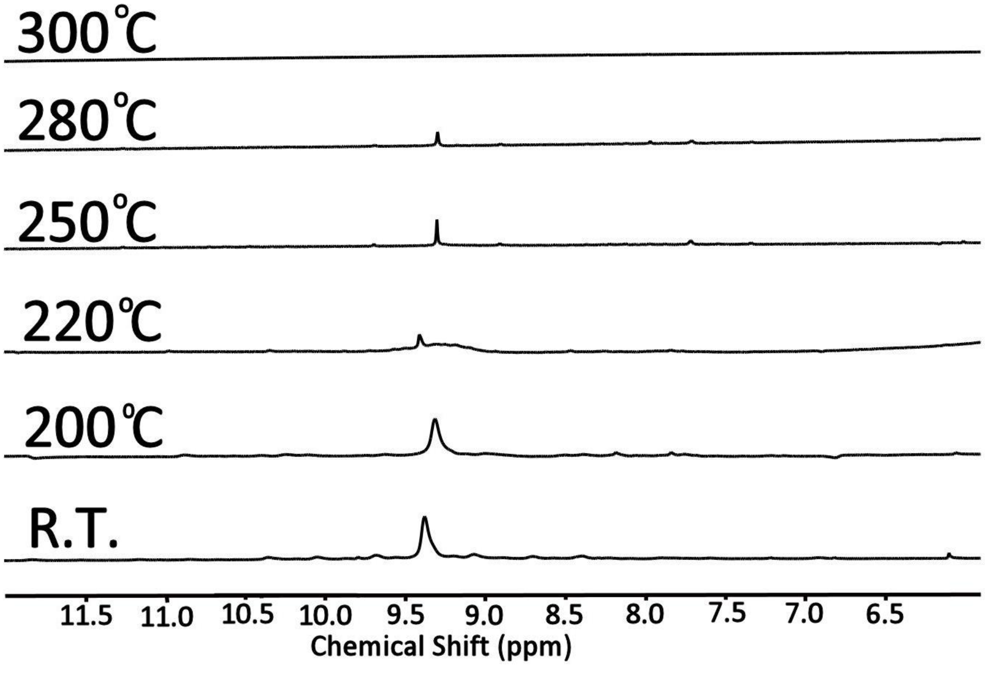

Fig S5. ^{1}H NMR with ^{31}P decoupled of the "Trojan Horse"-type cluster **1a** heated from R.T. to 300°C.

5. Mass Spectrometry data

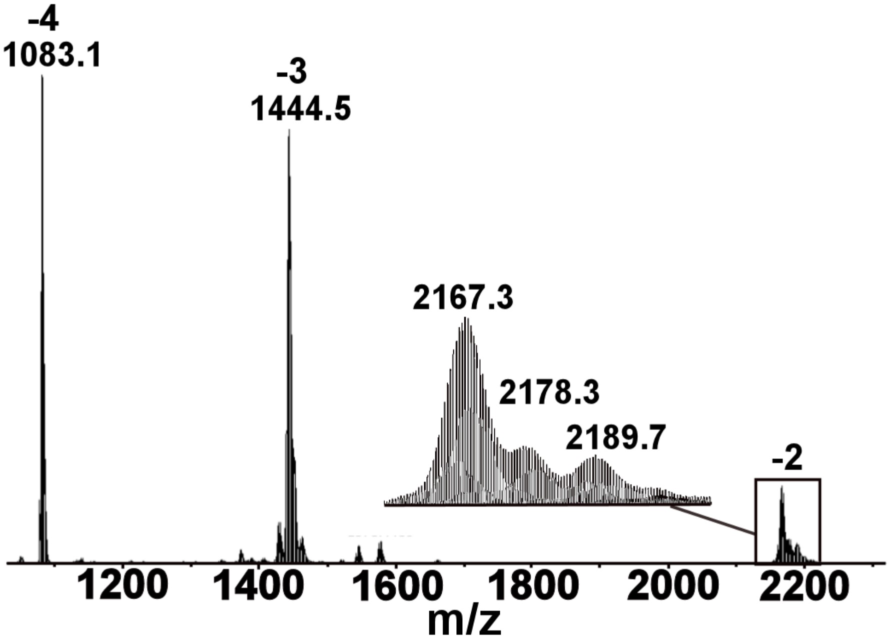

Figure S2. ESI-MS of Cluster **1a** in water/acetonitrile (5%:95%) solvent mixture..

Table S2. Mass spectral analysis of cluster **1a** in water/acetonitrile (5%:95%), with proposed formulae and charges..

m/z	***z***	**Assignment**
1083.1	4-	$\{W_{18}O_{54}(HPO_3)_2H_2\}$
1444.5	3-	$\{W_{18}O_{54}(HPO_3)_2H_1\}$
2167.3	2-	$\{W_{18}O_{54}(HPO_3)_2H_2\}$
2178.3	2-	$\{W_{18}O_{54}(HPO_3)_2H_1Na_1\}$
2189.7	2-	$\{W_{18}O_{54}(HPO_3)_2(C_2H_8N)_1H_1\}$

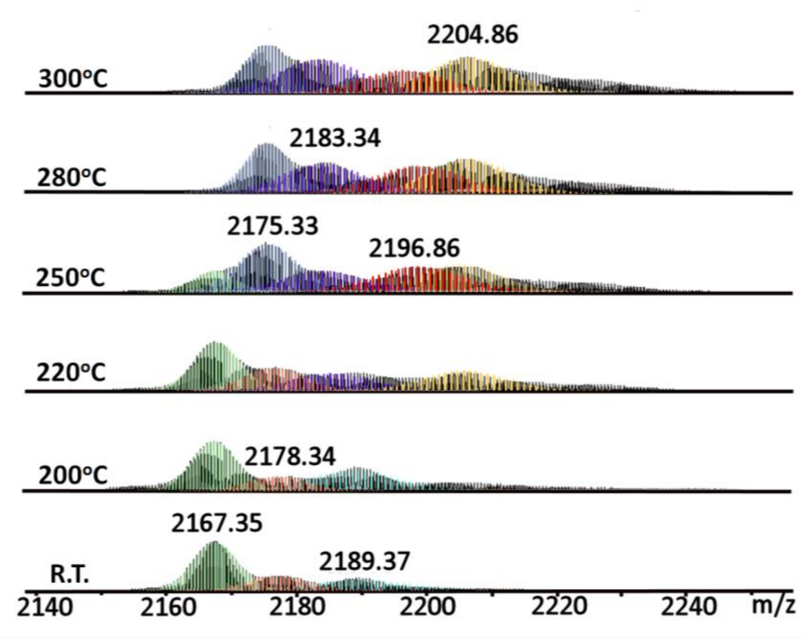

Fig S7. ESI-MS spectra of cluster **1a** in a water/acetonitrile (5%:95%) solvent mixture (charge 2-) heated from room temperature to 300°C.

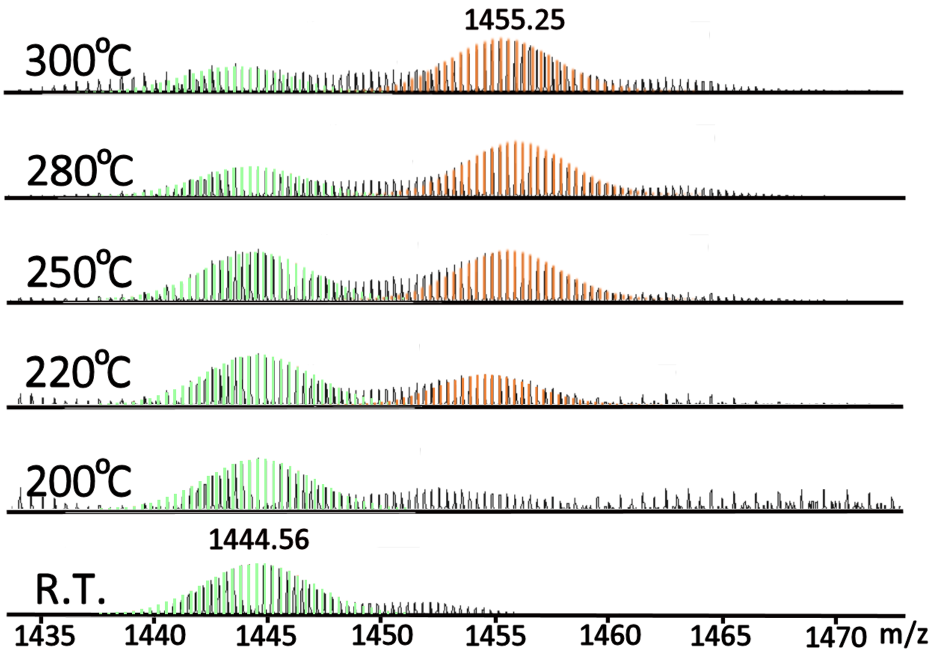

Fig S8. ESI-MS spectra of cluster **1a** in a water/acetonitrile (5%:95%) solvent mixture (charge 3-) heated from room temperature to 300°C.

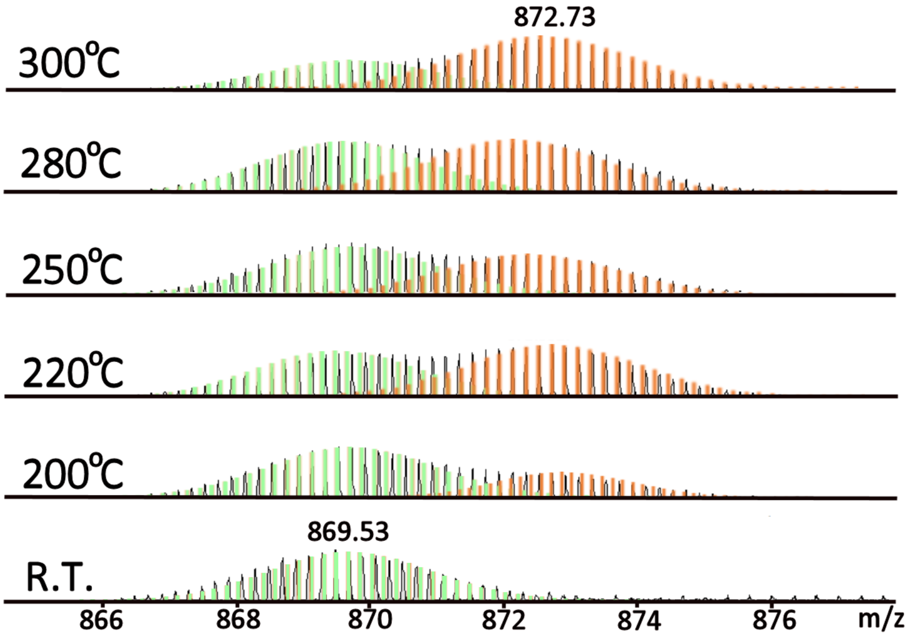

Fig S9. ESI-MS spectra of cluster **1a** in a water/acetonitrile (5%:95%) solvent mixture (charge 5-) heated from room temperature to 300°C.

Table S3. Mass spectral analysis of cluster **1a** heated to different temperatures, with proposed formulae and charges..

z	*m/z*	**Assignment**
-2	2167.35	$[W_{18}O_{54}(HPO_3)_2H_2]^{2-}$
	2178.34	$[W_{18}O_{54}(HPO_3)_2H_1Na_1]^{2-}$
	2189.37	$[W_{18}O_{54}(HPO_3)_2Na_2]^{2-}$
	2175.33	$[W_{18}O_{54}(HPO_3)(PO_4)H_3]^{2-}$
	2184.64	$[W_{18}O_{54}(PO_4)_2H_4]^{2-}$
	2196.86	$[W_{18}O_{54}(HPO_3)(PO_4)Na_2]^{2-}$
	2204.86	$[W_{18}O_{54}(PO_4)_2H_1(C_2H_8N)]^{2-}$
-3	1444.56	$[W_{18}O_{54}(HPO_3)_2H]^{3-}$
	1455.25	$[W_{18}O_{54}(PO_4)_2H_3]^{3-}$
-5	869.53	$[W_{18}O_{54}(HPO_3)_2(OH)]^{5-}$
	872.73	$[W_{18}O_{54}(PO_4)_2H]^{5-}$

6. EPR spectra

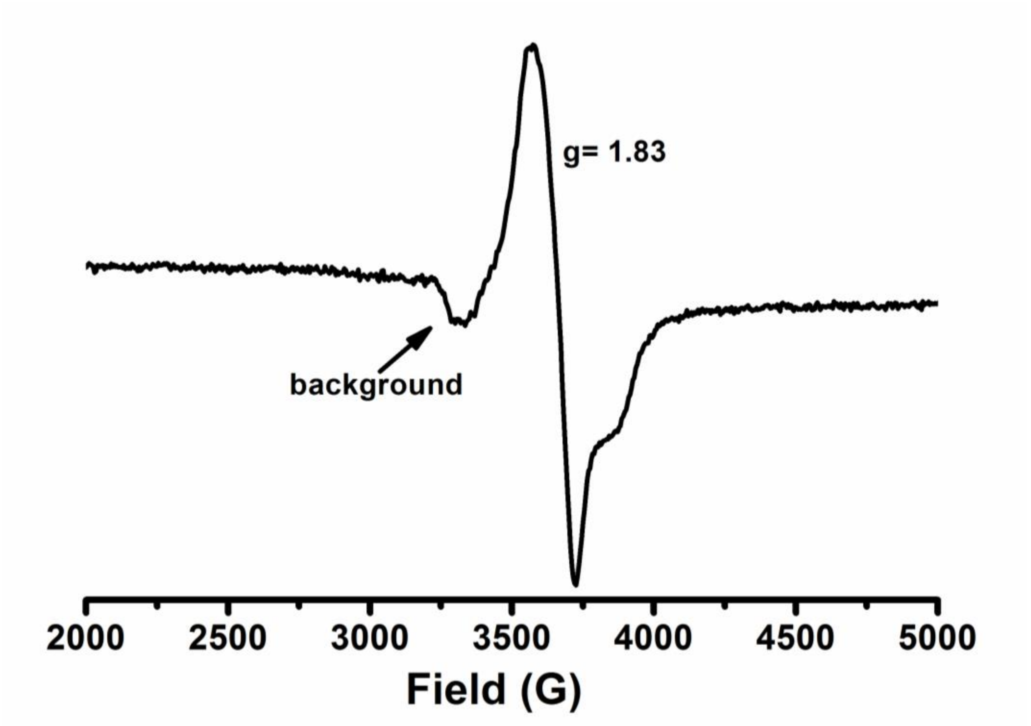

Fig S6. EPR carried out at **-**100 K for cluster **1a** heated to 220°C, showing a peak characteristic of a delocalized reduced tungsten (W^{V}), with an isotropic g-value of 1.83.

7. Infrared spectra

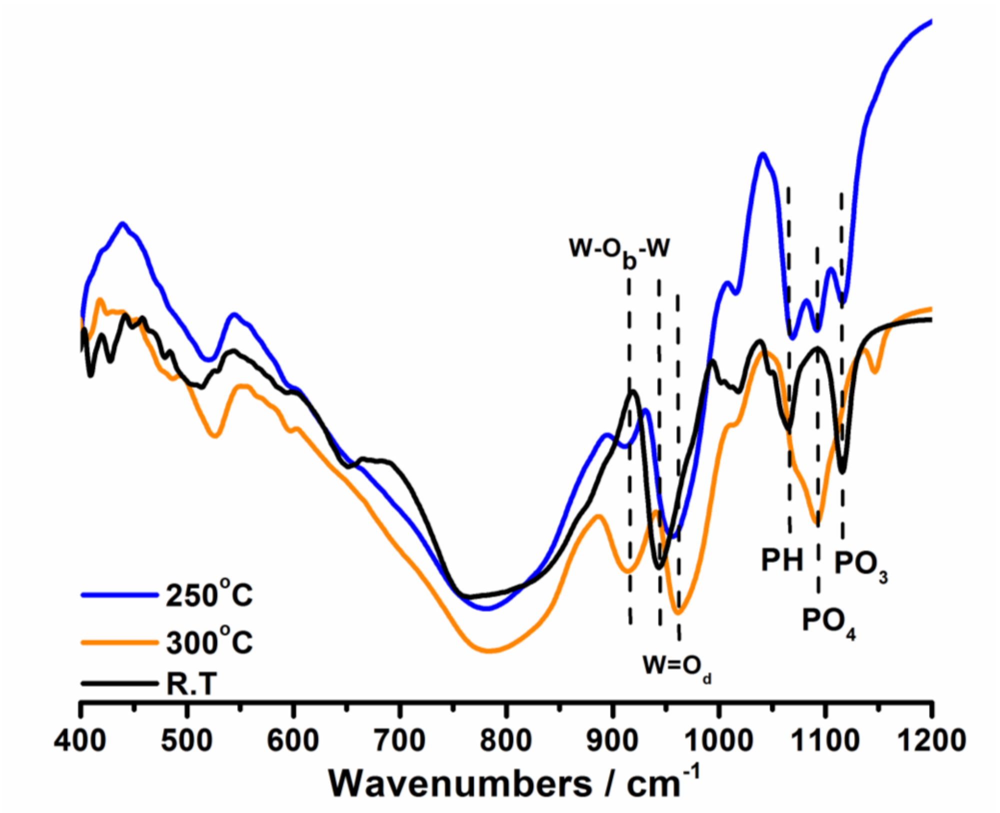

Fig S10. Infrared spectra ranging from 400 cm^{-1} to 1200 cm^{-1} of cluster **1** after different temperature treatments showing structural transformations. The blue shift of the (W=O$_d$) band at 943 cm^{-1} in both blue and yellow crystals indicated the gradual release of two water ligands during the thermal treatment; whereas the formation of characteristic W-O$_b$-W bond at 914 cm^{-1} in the yellow crystals confirmed the structural transformation to the classic Wells-Dawson cluster.[5,7] In addition, the P-H bending (1065 cm^{-1}) and P^{III}-O stretching (1115 cm^{-1})[6] in the colourless compounds was merged to P^{V}-O stretching at 1092 cm^{-1} [7] after they turned yellow, verifying the heat triggered embedded heteroanion oxidation process.

8. PXRD spectra

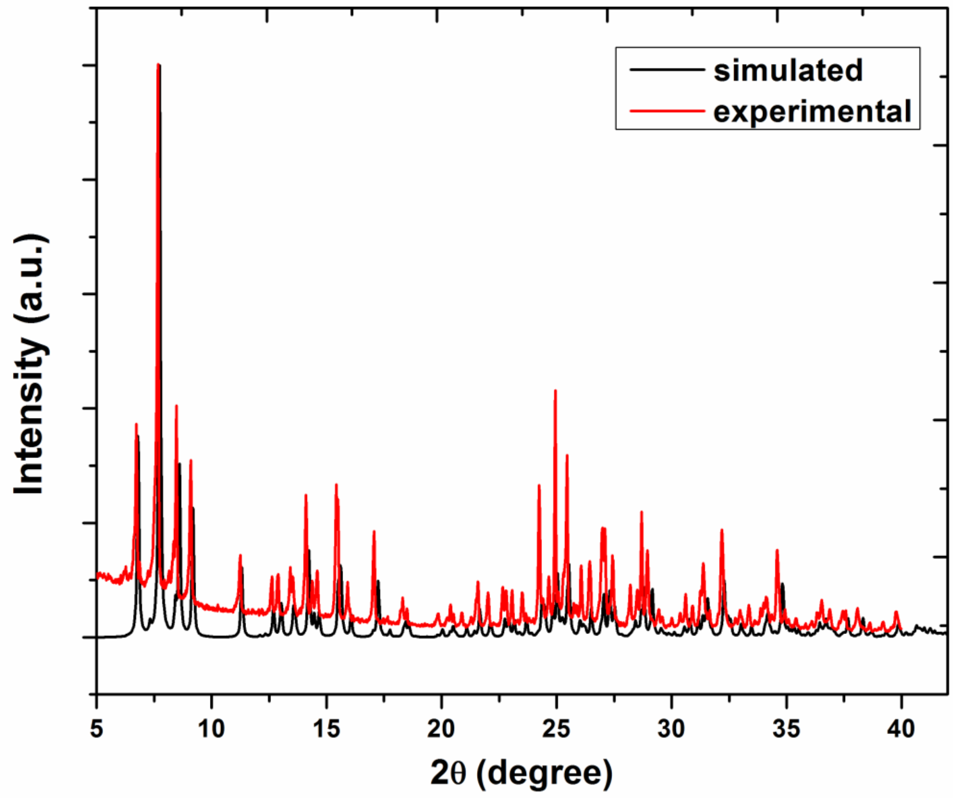

Figure S11. PXRD spectrum of cluster **1a** compared with the simulation pattern, showing the purity of the bulk compounds.

9. TGA and DSC

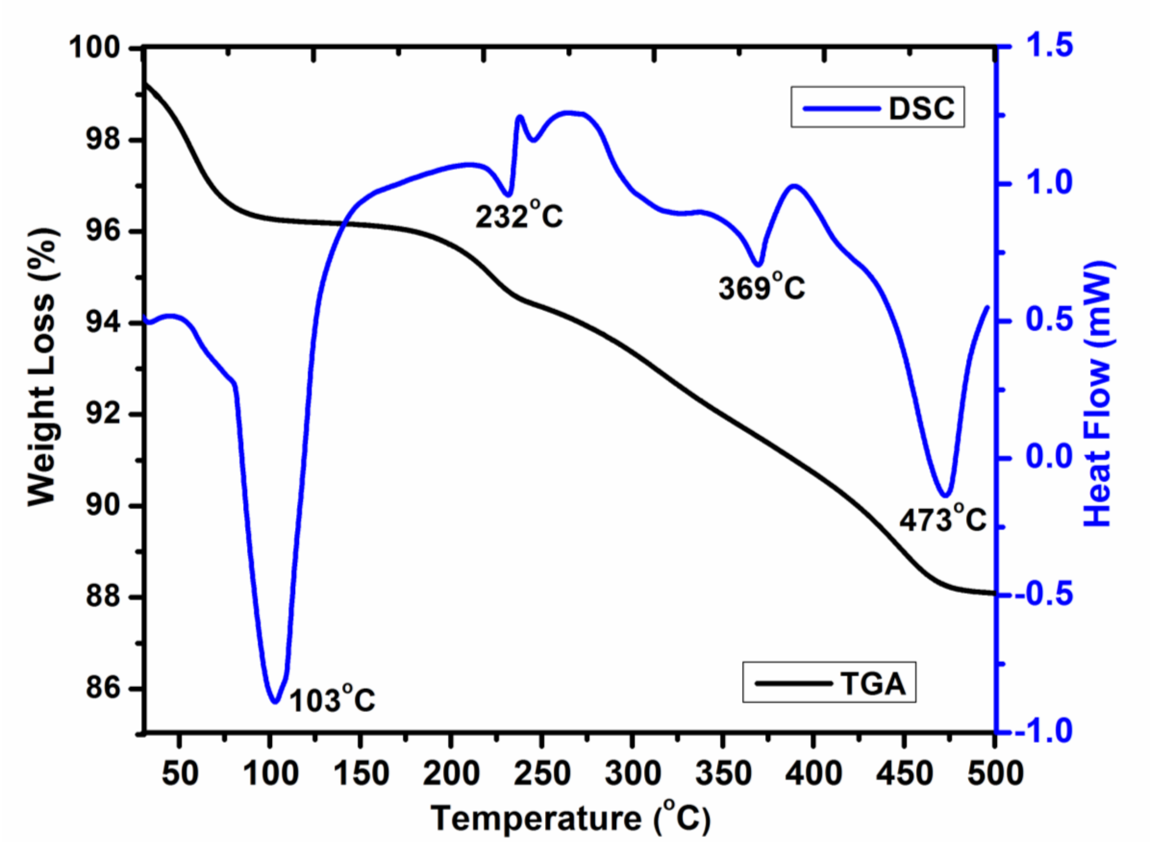

Figure S12. TGA and DSC of cluster **1a**.

10. Theoretical calculations

Density Functional Theory (DFT) Calculations The quantum chemical calculations performed for this study were done at the density functional theory (DFT) level. Fully converged geometries without symmetry restrictions were calculated using the B3LYP[8] method as implemented by the TURBOMOLE V6.3.1 package. TZVP basis set was used on all atoms.[9] To allow for solvation effects, the conductor-like screening model (COSMO) method was used.[10] The ionic radii of the atoms, which define the dimensions of the cavity surrounding the molecule, are chosen to be (in Å) 2.23 for W, 1.72 for O, 2.11 for P, 1.3 for H.

Table S4. Theoretical values of the total energy in Hartree (E_h), orbital Energies (in eV), HOMO-LUMO gap (eV).

Specie	*Compound*	E_h	E_{HOMO}	E_{LUMO}	$\Delta E_{H\text{-}L}$
$[W_{18}O_{56}(HP^{III}O_3)_2(H_2O)_2]^{8-}$	**1**	-6712.65	-7.18	-3.18	4.00
$[W_{18}O_{54}(HP^{III}O_3)_2]^{4-}$	**2a**	-6408.66	-8.15	-4.58	3.57
$[W_{18}O_{54}(HP^{III}O_3)(P^{V}O_4)]^{5-}$		-6483.52	-7.91	-4.41	3.50
$[W_{18}O_{54}(P^{V}O_4)_2]^{6-}$	**3a**	-6558.4	-7.8	-4.2	3.60

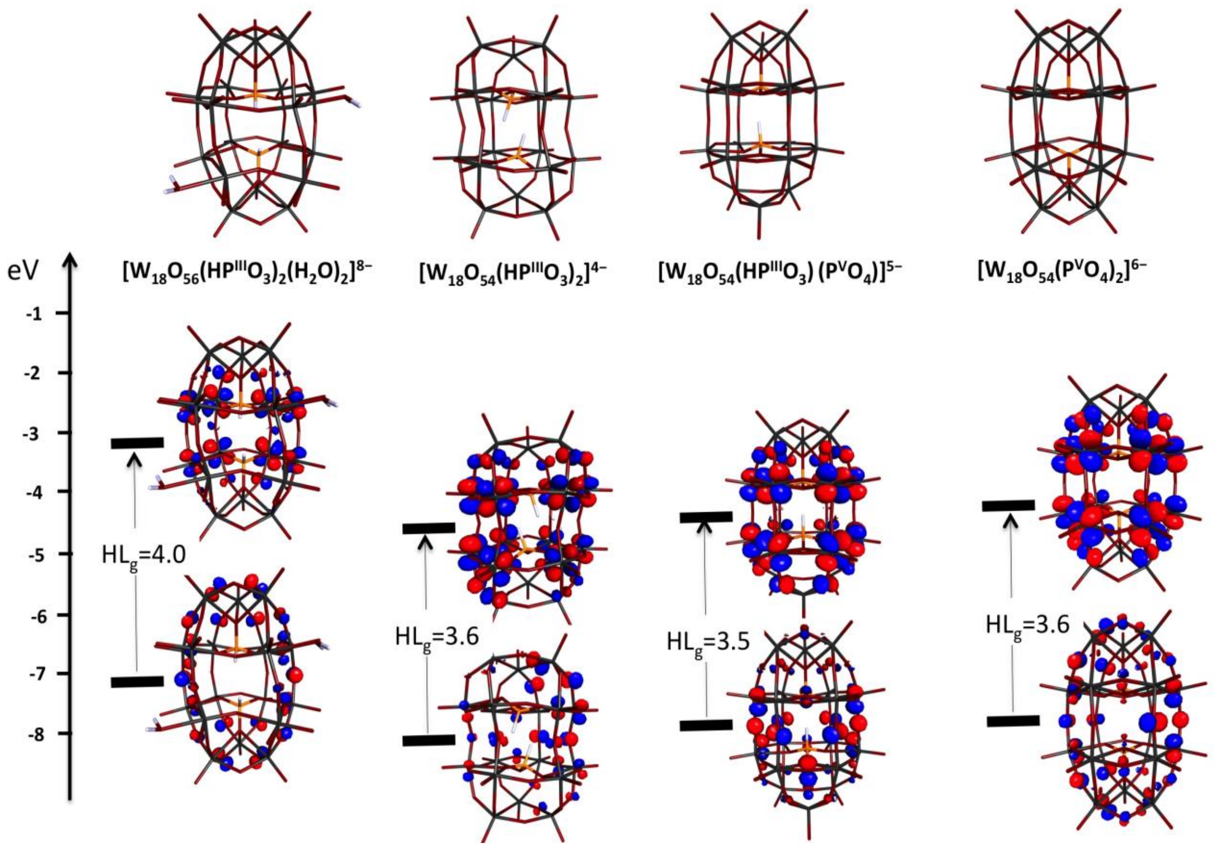

Figure S13. Frontier orbitals and HOMO-LUMO gap (HL_g) values in eV for the polyoxometalates in the present study $[W_{18}O_{56}(HP^{III}O_3)_2(H_2O)_2]^{8-}$ compound **1**, $[W_{18}O_{54}(HP^{III}O_3)_2]^{4-}$ compound **2a**, $[W_{18}O_{54}(HP^{III}O_3)(P^{V}O_4)]^{5-}$, $[W_{18}O_{54}(P^{V}O_4)_2]^{6-}$ compound **3a**. Note that the values for the HLg are in the same order of magnitude (~4eV), which suggests a relative high stability for all clusters.

11. References

1. G. Sheldrick, *ActaCrystallogr. Section A* **1990**, *46*, 467-473.
2. G. Sheldrick, *ActaCrystallogr. Section A* **2008**, *64*, 112-122.
3. L. Farrugia, *J. Appl. Crystallogr*. **1999**, *32*, 837-838.
4. R. C. Clark, J. S. Reid, *ActaCrystallogr. Sect. A* **1995**, *51*, 887-897.
5. I.-M. Mbomekalle, Y. W. Lu, B. Keita, L. Nadjo, *Inorg. Chem. Commun.* **2004**, 7, 86-90.
6. U. C. Chung, J. L. Mesa, J. L. Pizarro, V. Jubera, L. Lezama, M. I. Arriortua, T. Rojo, J. *Solid State Chem*. **2005**, 178, 2913-2921.
7. X. Qian, X. Tong, Q. Wu, Z. He, F. Cao and W. Yan, *Dalton Trans.* **2012**, 41, 9897-9900.
8. a) A. D. Becke, *Phys Rev A.* **1988**, 38, 3098-3100. b) R. H. Hertwig, W. Koch, *Chem. Phys. Lett* **1997**, 268, 345-351.
9. A. Schäfer, C. Huber, R. Ahlrichs, *J. Chem. Phys*. **1994**,100, 5829-5835.
10. a) A. Klamt, G. Schürmann, *J. Chem. Soc. Perkin Trans*. **1993**, 2, 799-805. b) A. Schäfer, A. Klamt, D. Sattel, J. C. W. Lohrenz, F. Eckert, *Phys. Chem. Chem. Phys.* **2000**, 2, 2187-2193.
